# Estimation of the *In Vivo* Release of Amiodarone From the Pharmacokinetics of Its Active Metabolite and Correlation With Its *In Vitro* Release

**DOI:** 10.3389/fphar.2020.621667

**Published:** 2021-02-15

**Authors:** Maytham Razaq Shleghm, Constantin Mircioiu, Victor A. Voicu, Ion Mircioiu, Valentina Anuta

**Affiliations:** ^1^ Carol Davila University of Medicine and Pharmacy, Bucharest, Romania; ^2^ Titu Maiorescu University, Bucharest, Romania

**Keywords:** amiodarone, desethylamiodarone, pharmacokinetics, in vivo release, in vitro in vivo correlation

## Abstract

Due to its very low water solubility and complex pharmacokinetics, a reliable point-to-point correlation of its *in vitro* release with its pharmacokinetics has not been achieved so far with amiodarone. The correlation of the *in vitro* dissolution of a drug with the pharmacokinetics of one of its metabolites was recently proposed by the authors of the article as an additional or alternative analysis to the usual *in vitro* correlations *in vivo*, mainly in the case of fast-absorbing drugs that have metabolites with a significant therapeutic effect. The model proposed by the authors considers that amiodarone has a slow dissolution, rapid absorption, and rapid metabolism, and before returning to the blood from other compartments, its pharmacokinetics is determined mainly by the kinetics of release in the intestine from the pharmaceutical formulation. Under these conditions, the rate of apparition of desethylamiodarone in the blood is a metric of the release of amiodarone in the intestinal fluid. Furthermore, it has been shown that such an estimated *in vivo* dissolution is similar, after time scaling, to the dissolution measured experimentally *in vitro*. Dissolution data of amiodarone and the pharmacokinetic data of its active metabolite desethylamiodarone were obtained in a bioequivalence study of 24 healthy volunteers. The elimination constant of the metabolite from plasma was estimated as the slope of the linear regression of logarithmically transformed data on the tail of plasma levels. Because the elimination of desethylamiodarone was shown to follow a monoexponential model, a Nelson–Wagner-type mass equilibrium model could be applied to calculate the time course of the “plasma metabolite fraction.” After Levi-type time scaling for imposing the *in vitro–in vivo* correlation, the problem became that of the correlation between *in vitro* dissolution time and *in vivo* dissolution time, which was proven to follow a square root model. To validate the model, evaluations were performed for the reference drug and test drug separately. In both cases, the scaled time for *in vivo* dissolution, t*, depended approximately linearly on the square root of the *in vitro* dissolution time *t*, with the two regression lines being practically parallel.

## Introduction

Amiodarone (AMD) has been shown to have variable oral bioavailability (20–80%). After absorption, AMD undergoes extensive metabolism, is distributed in the blood, lipids, and in deep compartments, and undergoes enterohepatic circulation (Holt et al., 1983). Metabolism includes a first and second N-dealkylation, an O-dealkylation as well as a first and second hydroxylation. Glucuronidation was also highlighted. The major and active metabolite is desethylamiodarone (DAMD) (Berger and Harris, 1986; Deng et al., 2015).

Concentrations in the myocardium have been shown to be 35 times higher than in the plasma (Djiane et al., 1984). The pharmacokinetic model is usually considered to be multicompartmental, including the central compartment, the lipid compartment, and a deep compartment (Freedman and Somberg, 1991).

*In vitro–in vivo* correlations (IVIVCs) are correlations between *in vitro* dissolution data and *in vivo* release kinetics, estimated by the deconvolution of pharmacokinetic IVIVCs were constantly recommended by regulatory authorities in the last decades when developing extended-release formulations (US Food and Drug Administration, 1997a; US Food and Drug Administration, 1997b; European Medicines Agency, 2014a; European Medicines Agency, 2014b). The correlation can be good and even linear (Humbert et al., 1994; Eddington et al., 1998; Emami 2006) or nonlinear [Lake et al., 1999; Varshosaz et al., 2000; Rao et al., 2001; Al-Behaisi et al., 2002], or even obscure (Eddington et al., 1998; Mircioiu et al., 2005; Meyer et al., 1998; Mircioiu et al., 2018). Complex models were proposed in cases of nonlinearity (Polli et al., 1996; Dunne et al., 1997; Dunne et al., 1999), but the number of parameters of models is higher, and the fitting algorithms are more unstable (Sandulovici et al., 2009; Tvrdonova et al., 2009).

A major complication occurs when *in vitro* dissolution is forced to be rapid and complete by the addition of surfactants in the dissolution medium, in which case the need arises to scale the time before correlation (Levy et al., 1967).

A first correlation between amiodarone *in vitro* dissolution and its *in vivo* dissolution estimated by the deconvolution of plasma levels was performed by Emami; however, as a consequence of very complex pharmacokinetics, results were reliable only for types B and C correlations.

The present article attempts to apply a recent method (Mircioiu et al., 2019a) of correlation between the dissolution of the parent drug and the pharmacokinetics of one of its metabolites, to the correlation between the *in vitro* dissolution of amiodarone and the rate of plasma desethyl active metabolite of amiodarone, based on data from a bioequivalence study.

## Materials and Methods

### *In Vitro* Dissolution

The release of amiodarone from six tablets was evaluated using a USP 2 dissolution apparatus (DT 800 Erweka GmbH) at 100 rpm. The dissolution medium was sodium lauryl sulfate 10 g/L in ultrapure water (1,000 ml). Samples of 5 ± 0.1 ml were collected at 5, 15, 30, 45, and 60 min and subsequently replaced with an equal volume of medium. AMD concentrations were determined at 242 nm on a V-530 UV-VIS spectrophotometer (JASCO Ltd., Tokyo, Japan).

### Clinical Trial

*In vivo* data were obtained in a bioequivalence study by comparing a tested formulation (T) with reference (R) Cordarone 200 mg, Sanofi Synthelabo. The study was approved by the Romanian National Medicines Agency and Ethics Committee of the Army Center for Medical Research.

Venous blood samples (5 ml) were collected into heparinized tubes through a catheter inserted in the antecubital vein before (time 0) and at 1, 1.5, 2, 3, 3.5, 4, 4.5, 5, 5.5, 6, 6.5, 7, 7.5, 8, 9, 10, 12, 24, 48, 72, 96, and 120 h. Blood samples were centrifuged at 5°C for 6 min at ∼3,000 rpm. Plasma was immediately frozen and stored at −30°C until analysis.

### Bioanalytical Method

#### Sample Treatment

Plasma samples (1,000 µL) were transferred to 10 ml disposable polypropylene tubes, to which 50 µL internal standard (IS) solution (20 µg/ml fenofibrate in methanol), 500 µL pH 4.5 phosphate buffer, and 4 ml methyl tert-butyl ether were added. The tubes were vortex mixed for 10 min and then centrifuged for 10 min at 4,000 rpm. Of the organic layer, 3 ml were retaken and evaporated to dryness at 40°C under a gentle nitrogen steam. The sample was reconstituted into 200 μL of mobile phase. Of each sample, 100 µL were injected into the chromatographic column.

#### Chromatographic Analysis

The chromatographic analyses were performed on a Waters liquid chromatographic system (Milford, MA 01757, United States) consisting of a 600E quaternary gradient system, an AF model in line degasser, 486 UV-VIS tunable absorbance detector, and a 717 plus auto sampler. Empower Pro software (Waters, Milford, MA 01757, United States) was used to control the system and acquire and process data. The UV detector was set at 242 nm. A 15 cm × 4.6 mm i.d Microsorb-MV C18 column (Varian, Crawley, United Kingdom) and a guard column packed with C18 were used for separation. The mobile phase consisted of a phosphate buffer solution containing 7 mM Na_2_HPO_4_ and 11 mM KH_2_PO_4_, adjusted to pH 4.5 (Solvent A) and a 1:1 (v:v) acetonitrile methanol mixture (Solvent B), and delivered in a 20:80 (v:v) ratio. The mobile phase was prepared daily, filtered, and degassed before use. The flow rate was 1.0 ml/min, and all work was carried out at 40°C.

The method was validated in accordance with the bioanalytical method validation guidelines of the FDA, including linearity, limits of quantification, selectivity, accuracy, precision, recovery, dilution effects, and stability. The specificity was evaluated related to interferences from the endogenous matrix components of drug-free plasma samples of six different origins. The calibration curves of AMD and DAMD were constructed in the range in the range 20–1,000 ng/ml for both AMD and DAMD, by plotting the ratios between their peak areas and IS peak areas vs. concentration (ng/ml), using data obtained from triplicate analysis of the calibration standard solution. The lower limit of quantification (LLOQ) was set as the lowest concentration on the calibration curve. Within-run and between-run precision and accuracy were estimated by analyzing five replicates of the LLOQ and quality control (QC) samples in a single analytical run and on five consecutive days, respectively. The absolute recovery of AMD and DAMD was determined using five replicates of the three concentration level QC samples and was determined to be 74% for AMD and 97% for DAMD. Benchtop, extract, stock solution, freeze-and-thaw, long-term, and post-preparative stability studies were also performed to evaluate the stability of both analytes.

### Statistical Analysis

Pharmacokinetics parameters area under curve ($AUC_{0-\infty }$) and maximum concentration (C_max_) were considered as random variables with the following structure (Chow et al., 1997):

Y_ijk_ = μ + S_ik_ + P_j_ + F_(j,k)_ + C_(j-1,k)_ + e_ijk_,

where μ = the overall mean, i = index for subject, i = 1, n_k_, j = index for period, k = index for sequence, F_(j,k)_ = the direct fix effect of the formulation in the kth sequence which is administered at the jth period, C_(j-1,k_ = the fixed first-order carryover effect of the formulation in the kth sequence which is administered at the (j-1)th period, where C_(0,k)_ = 0 and ΣC_(j-1,k)_ = 0, and e_ijk_ = the within-subject random error in observing Y_ijk_.

All parameters were evaluated by analyses of variance to determine statistically significant (α = 0.05) differences between the drug formulations using the program Kinetica, version 4.2. InnaPhase Corporation.

To demonstrate bioequivalence, the 90% confidence intervals for AMD (DAMD) test/reference ratios of AUC_0-τ_ and AUC_0-∞_ were shown to lie within the 80–125% interval.

### Modeling

#### Modeling of *In Vitro* Release Kinetics Data

*In vitro* dissolution data were modeled using a square root law and a power law model, used in linear forms, as previously described (Mircioiu et al., 2013).

The law of square root can result from a phenomenological model that involves the diffusion of the drug into the solvent that penetrates the matrix of the pharmaceutical formulation (Higuchi model) or from a model that considers release from the pharmaceutical formulation as an infinite reservoir across the interfaces with the solvent in a long diffusion path (Mircioiu et al., 2019b):

$$r\left(t\right)=k\sqrt{t},$$

where *r(t)* is the ratio of cumulated released substance at the moment t. It should be noted that *r(t)* is sometimes written in the form *r*(*t*) = *M*(*t*)/*M*_∞_, where *M*_∞_ is the amount released at infinity; however, in all cases, this is not the total amount of diffusing component. In case of nanosystems, for example, the release most frequently involves only a part of the active substance, which we can consider as the “available fraction for release,” with another part of it remaining sequestered. Whatever the case, in practice, in most cases, the experimentally determined quantity tends to reach a saturation value. If this value remains constant for a sufficient period of time, it is reliable to consider it as *M*_∞_.

Power law is an empirical law which combines two release kinetics as a result of the diffusion and the erosion of a matrix, is linearized in the form

ln(r)=ln⁡k+n⁡ln(t),

and is known in case of release from pharmaceutical formulations, under the name Peppas law (Peppas 1985).

#### Modeling of AMD and DAMD Pharmacokinetics

Analysis of time evolution of plasma levels of AMD and DAMD and estimation of the pharmacokinetic parameters was performed by both non-compartmental and compartmental methods, based on the data obtained in the 0–120 h time interval.

There were estimated partial and cumulated areas under curves. It was tested if, after logarithmic transformation, a good regression line on the tail of the curve was obtained, in order to define an elimination constant. Mono- and bicompartmental modeling was tested for AMD and DAMD pharmacokinetics.

#### Pharmacokinetic Model for Dissolution, Absorption, and Metabolism of AMD and Formation and Elimination of DAMD

Amiodarone, a lipophilic drug (logP = 7.24), undergoes substantial metabolism, being classified as BDDCS (biopharmaceutics drug disposition classification system) Class 2 compounds (Wu and Benet, 2005).

The hypothesis of this article, presented previously by the authors (Mircioiu et al., 2019a), was that if the absorption and metabolism can be assumed to be rapid, then the apparition of metabolite in plasma $FRAp^{dAMD}$ (*t*) could be considered an estimation of the absorption of the parent drug from the intestine *FRA*(*t*_*i*_). Based on this hypothesis, a correlation between *in vitro* dissolution and the *in vivo* pharmacokinetics of metabolites would be expected, which was indeed found in the case of diltiazem.

Because the pharmacokinetics was measured after a single dose, the return from the “deep compartment,” where accumulation occurs over time, was neglected. Furthermore, because metabolites occur at the same time as plasma AMD, metabolism is considered a rapid process.

Consequently, the slowest, rate-determining step for the chain of kinetics leading to the apparition of metabolite in plasma remains the release kinetics of the parent drug in the gastrointestinal tract.

Again, because AMD is lipophilic, the rate of transfer from the blood to the lipid compartment is higher than that of reverse transport; the return of AMD to the blood may be neglected, and the transfer from blood to lipids will become a component of the elimination of the parent drug.

**SCHEME 1:**
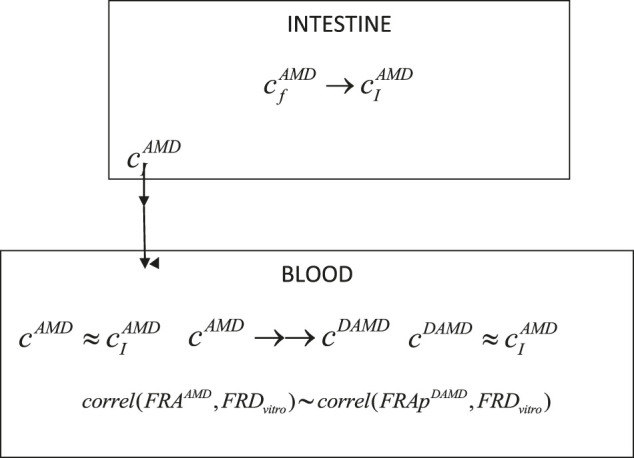
Schematic representation of the main processes involved in the pharmacokinetics of AMD and its DAMD metabolite.

Consequently, in a simplified one-compartment model for DAMD, it was considered only two processes, corresponding to the appearance of the metabolite in the blood and its total elimination (Scheme 1).

Where $c_{f}^{AMD}$ is the concentration in the tablet formulation, *c* ^*AMD*^ and *c* ^*dAMD*^ are, respectively, the concentrations of the parent drug and metabolite in blood compartment.

→ represents a slow process and →→ a rapid process, *FRA* ^*AMD*^ is the absorption fraction of AMD, $FRAp^{dAMD}$ is the fraction of apparition of DAMD in blood, *FRD* is the dissolution fraction, and *correl* denotes correlation.

#### Calculation of fraction of apparition of DAMD in plasma

A modified, Wagner–Nelson-type equation (Mircioiu et al., 2019a) was applied for the calculation of the “fraction of apparition” in plasma of the metabolite (*FRAp*):

$$FRAp^{dAMD}\left(t_{i}\right)=\frac{c^{dAMD}\left(t_{i}\right)+\int_{0}^{t_{i}}k_{e}^{dAMD}c^{dAMD}dt}{\int_{0}^{\infty }k_{e}^{dAMD}c^{dAMD}dt},$$

where *FRAp* ^*DAMD*^ is the fraction of the apparition of the metabolized drug at time *t*_*i*_, *c* ^*dAMD*^(*t*_*i*_) is the plasma concentration of the metabolite at time *t*_*i*_, and $k_{e}^{dAMD}$ denotes the apparent elimination rate constant.

The elimination rate constant was estimated as the slope of linear regression of the last points of the logarithmic transformed data. Integrals were approximated by areas under plasma levels of DAMD.

The model could actually be much more general. In the case of compounds subject to extended metabolism (BDDCS classes 1 and 2 compounds), because the rate of absorption and metabolism are usually high, the rate of occurrence of metabolites in the plasma is determined by the rate and extent of the parent drug release from the pharmaceutical formulation.

## Results and Disscussion

### *In Vitro* Dissolution of Amiodarone

**FIGURE 1:**
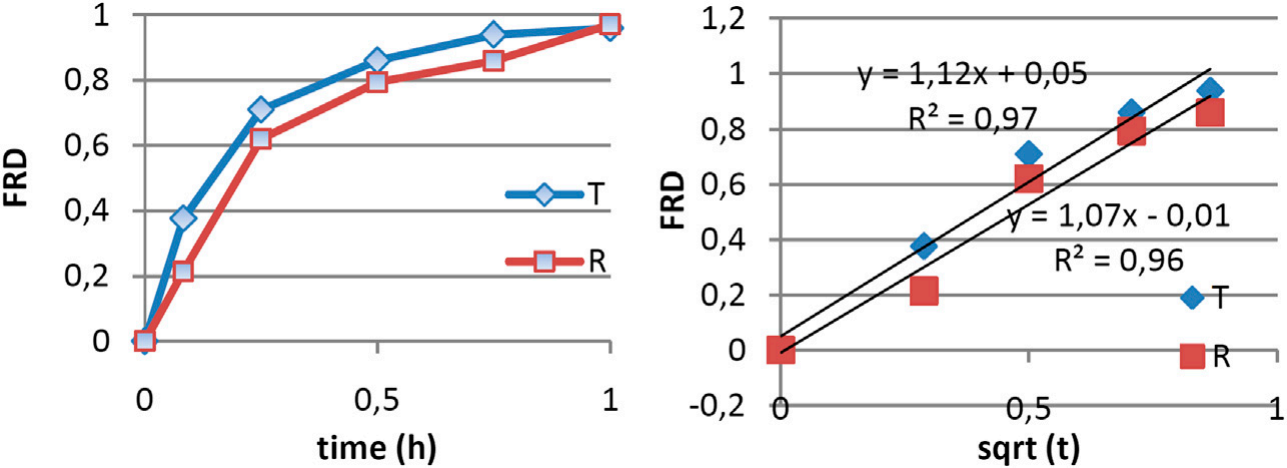
*In vitro* release data of AMD and modeling using the square law.

Since amiodarone is lipophilic (logP 7.24 ) [Amiodarone DrugBanK], its dissolution in water is very low, meaning that it is necessary to add surface-active agents in dissolution medium. The FDA recommends sodium lauryl sulfate (SLS) 1% or Tween 80 1% (accessdata.fda). In these conditions, the dissolution of AMD was rapid, being complete within 1 h in all cases. The mean amiodarone dissolution profiles are presented in Figure 1A.

### Modeling of the *In Vitro* Release

Dissolution is forced by the addition of a high concentration of surfactant in the release medium, which is a good test for quality control, but dissolution in the presence of great concentrations of surface-active agents is not biorelevant (Preda et al., 2012; Mircioiu et al., 2013).

The modeling of release kinetics was performed using both the square root and power law model. It appeared that both models work well enough. Correlation coefficient was higher in the case of the power law, but the number of points approximated by the square root law was greater. Fitting with the square root law for tested and reference drug are presented in Figure 1.

### Pharmacokinetics of AMD and DAMD

**FIGURE 2:**
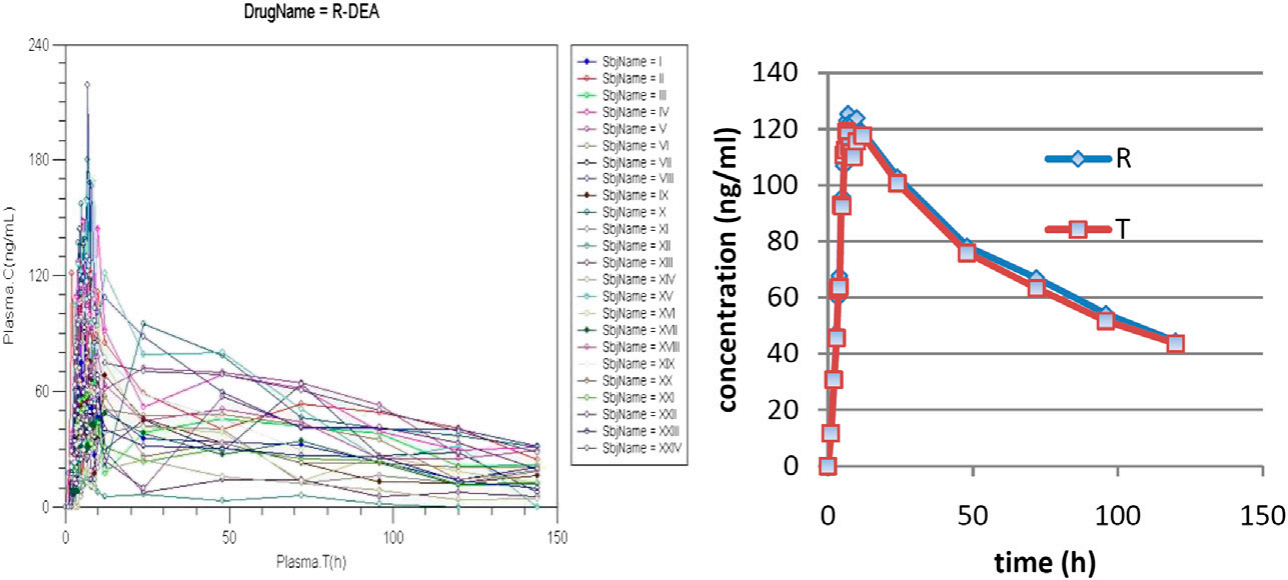
Plasma levels of DAMD.

Individual pharmacokinetics curves for AMD and mean curves for AMD and DAMD, for the reference (R) and tested (T)formulations are presented in Figure 2.

There is a great variability of concentrations between subjects from 12 h, but it is, at the same time, to note that the tails of curves are approximately parallel, suggesting a common pattern for elimination in all subjects. AMD has unpredictable absorption and therefore bioavailability (Martin Algara et al., 1994).

In the first phase, a rapid decrease in plasma levels appeared, with lipids and deep compartments becoming depots for both AMD and DAMD. Later, both of them return to the central compartment, and a long and variable terminal elimination half-life appears (Holt et al., 1983).

A naked eye analysis suggests that the formulations are bioequivalent. Mean pharmacokinetic parameters and 90% confidence intervals for mean ratios μAUCTμAUCR and μCmaxTμCmaxR presented in Table 1 confirmed the bioequivalence.

**TABLE 1:** Pharmacokinetic parameters of DAMD, non-compartmental analysis.

Pharmacokinetic parameter	Amiodarone (tested)	Cordarone	90% confidence interval
C_max_ (ng/ml)	103.6 ± 44.8	105.5 ± 48.3	88–111
T_max_ (h)	8.2 ± 8.8	6.1 ± 2.8	
AUC_0–120 h_	4507.9 ± 2043.1	4880.8 ± 2159.6	
$AUC_{0-\infty }$	6142.8 ± 2836	6783.2 ± 3154	84–104

### Calculation of the *In Vivo* Dissolution/Absorption Fraction as a Function of Time by Deconvolution of DAMD Plasma Levels

As the formulations proved to be bioequivalent in spite of their high variability, starting from AUC and Cmax, a first analysis was performed on the entire set of data in the study (joint, reference and tested, 48 curves).

To apply the mass balance of the Wagner–Nelson type in the calculation of the fraction of drug absorbed and, in our case, the fraction of AMD dissolved *in vivo*, the elimination constants for AMD and DAMD were estimated.

Half-time was not well defined in the case of AMD, with the result depending on the interval selected on the tail of the plasma level curves. Three, very different values were obtained: 7 h in the 7–12 h interval, 23 h in the 12–48 h interval, and 77 h in the 48–120 h interval. In the label of the AMD reference drug, a half-time of 53 days is reported. This evolution is a result of the distribution in lipids and enterohepatic circulation, as well as returning AMD back to the central compartment from the accumulations in lipid and deep compartments.

Comparative *in vitro* and *in vivo* evaluations of three tablet formulations of amiodarone in healthy subjects were previously reported by Emami (2010). He considered the last sampling time for *in vitro* dissolution, 120 min, and the *in vivo* time points of up to 18 h. He applied a time scale, following the FDA recommendation: “Time scaling may be used as long as the time scaling factor is the same for all formulations.” His conclusion was that “a point-to-point acceptable and reliable correlation was not achieved” and “dissolution data could be used only for routine and in-process quality control of amiodarone tablet formulations.”

**FIGURE 3:**
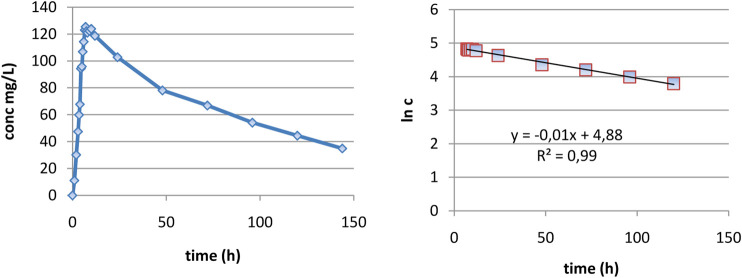
Evaluation of the elimination part of plasma level curves: **(A)** linear representation and **(B)** logarithmic representation.

In the case of DAMD, as can be seen in Figure 3A, curves are very smooth and elimination appears to be well described as monoexponential. In the interval 7–120 h, the logarithm of concentration was excellent linearly correlated with the time (Figure 3B), proving really a monoexponential behavior, and it was possible to calculate the half-time of DAMD, with a value of 70 h being obtained.

**FIGURE 4:**
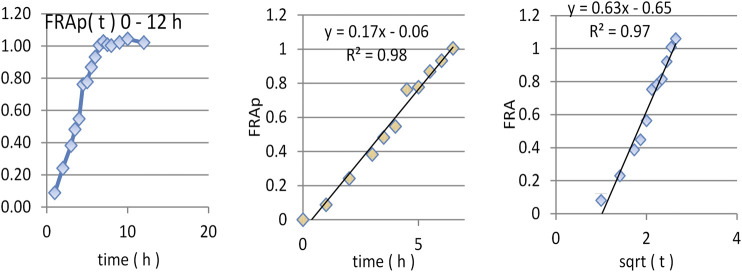
Dependence of FRAp(t) on time **(A**,**B)** and square root of time **(C)**.

By introducing this value in the proposed deconvolution formula and making the calculation, as can be seen in Figure 4A, a standard “absorption fraction” was obtained: a continuous smooth function increase followed by a saturation portion, at the limit value 1. This is a good result since, in the case of AMD, the curve had several maxima and even maxima greater than 1.

As the pharmacokinetic model supposes that the apparition of DAMD in plasma equals the release of AMD *in vivo*, an FRAp dependence on time similar to the model of dissolution kinetics *in vitro* could be expected. A naked eye examination suggests a linear model. A good fit of FRA as a function of the square root of time was also was obtained.

The linear correlation is just slightly better, but the small lag time appeared in the square root of time scale; this was a good result since absorption and metabolism are not instantaneous.

### *In Vitro*–*In Vivo* Dissolution Time Scaling

Following the low solubility of AMD and the small volume of GI liquids, dissolution had reason to be slow and limited. Release is also influenced by the secretion of bile salts and lecithine (Pahomi et al., 2012). Release in 1 h obtained in conditions of compendium test is a forced release. It is expected that *in vivo* release is much slower.

n circumstances of the model, the apparition of metabolite in plasma is correlated with *in vivo* dissolution of the parent drug. In order to correlate the *in vitro* dissolution fraction with the *in vivo* appearance of metabolite, time scaling was performed. Time in the interval 0–60 min, corresponding to *in vitro* dissolution, was transformed in time t* in the interval 0–7 h.

**FIGURE 5:**
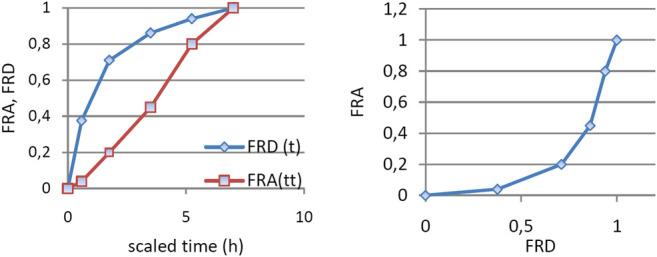
**(A)** FRA and FRD as functions of time scaled using a constant factor and **(B)** dependence of FRA of FRD.

An exponential dependence of the FRA on FRD (Figure 5) is difficult to interpret. Usually, a linear dependence is desired.

It has been used in the literature as a factor for time transformation in the ratio T50% *in vivo* dissolution/T50% *in vivo* dissolution (Yuen et al., 1983). In our case, this would give a factor of approximately 10, which is too much since FRA attains the value 1 at 7 h. Such an approach is imposed when the value 1 is reached asymptotically, not sudden, as in our case. However, following the complexity of the release *in vivo* and absorption, such an approach remains a rough approximation, which leaves out a lot of information deduced from dissolution and blood level profiles. This is also a common feature of other proposed methods, based on statistical moment analysis (Tanigawara et al., 1982).

The application of a constant factor, the same for different formulations, is an ideal method. Unfortunately, it is expected that in different segments of the GI tract, the influence on the dissolution rate is different and the application of a single factor leads to a too rough approximation (Cardot and Davit, 2012; Marvola et al., 2004; Hemmingsen et al., 2011).

**FIGURE 6:**
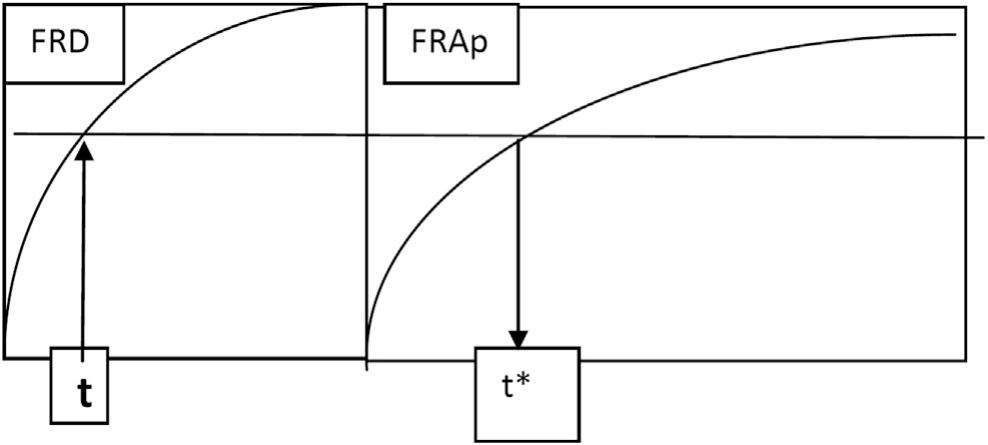
Graphical presentation of the time scaling t–t* algorithm.

The alternative method used in this article was to look for a transformation of time (Figure 6) which leads to a linear dependence between FRA(t*) and FRD(t*).

Transformation of time was performed, as can be seen in Figure 3. Time t was transformed in time t*, for which FRA(t*) = FRD(t). In fact, this is a method to obtain a Levy plot.

After Levi-type time scaling for imposing the *in vitro–in vivo* correlation, the problem became that of the correlation between *in vitro* dissolution time *t*, and *in vivo* dissolution time t*, which was proven to follow a square root model.

**FIGURE 7:**
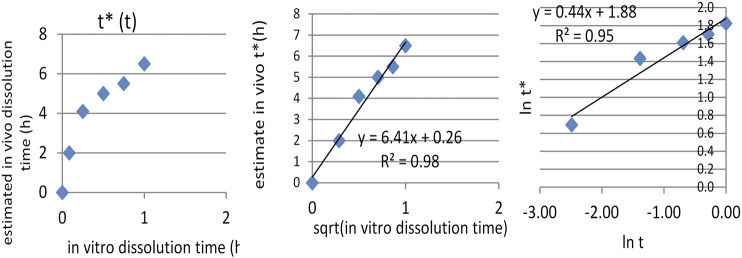
Dependence of t* on t **(A)**, of square root of t **(B)**.

**FIGURE 8:**
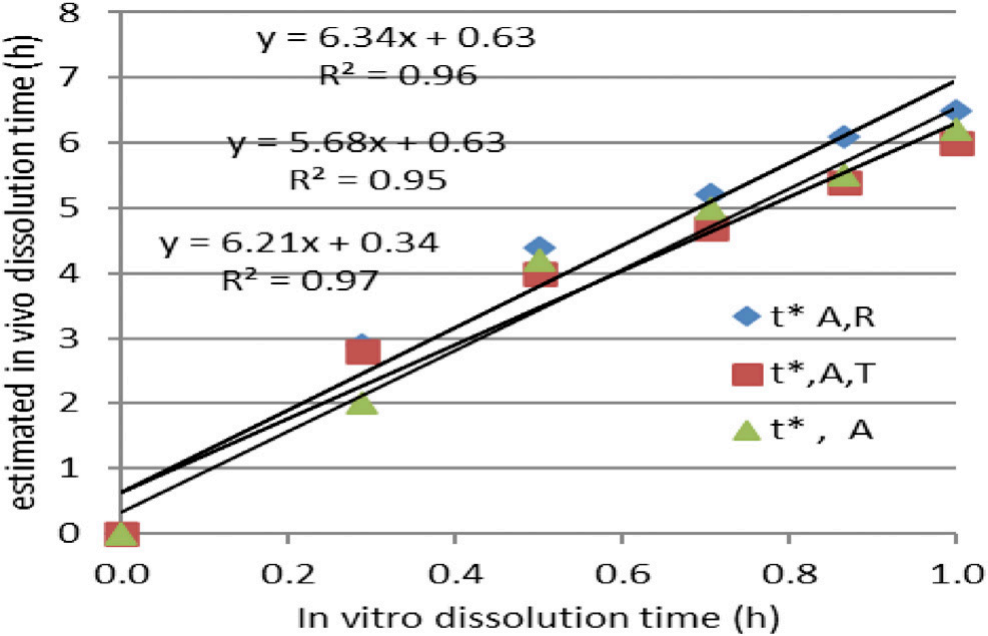
Modeling the dependency t*(*t*) as a square root–type law for the reference drug, tested drug, and joint data.

IA function t*(*t*) was obtained, as can be seen in Figure 7A. The dependence t* on the square root of *t* (Figure 7C) seemed to be reliable. This function has a much more mechanistic resonance. Although usually applied for describing the release kinetics data, it proved to also be applicable in the case of AMD tablets in both our experiments (Figure 8). This represents a more general phenomenon: release from infinite reservoirs, similar to thermostats in heat transfer theory (Mircioiu et al., 2019b).

## Conclusion

In the case of lipophilic drugs, due to slow dissolution, rapid absorption, and rapid metabolism, the pharmacokinetics of both the parent drug and metabolites before the return of the drug from other compartments in the blood is mainly determined by the kinetics of release in the intestine from the pharmaceutical formulation.

For long-life lipophilic drugs, as shown for DMA, it is possible to estimate the absorption fraction of the parent drug from the simpler pharmacokinetics of the metabolite, in which case it is possible to calculate an elimination constant.

The similarity between *in vitro* dissolution and the *in vivo* estimated dissolution models as well as the similar dependence of scaled time on *in vitro* time in the case of bioequivalent formulations can be considered a validation of the metabolite approach of the *in vitro*–*in vivo* correlation model.

## Data Availability

The raw data supporting the conclusions of this article will be made available by the authors, without undue reservation.
